# Reliability of Contractile Properties of the Knee Extensor Muscles in Individuals with Post-Polio Syndrome

**DOI:** 10.1371/journal.pone.0101660

**Published:** 2014-07-14

**Authors:** Eric L. Voorn, Merel A. Brehm, Anita Beelen, Arnold de Haan, Frans Nollet, Karin H. L. Gerrits

**Affiliations:** 1 Department of Rehabilitation, Academic Medical Center, University of Amsterdam, Amsterdam, The Netherlands; 2 MOVE Research Institute Amsterdam, Faculty of Human Movement Sciences, VU University Amsterdam, Amsterdam, The Netherlands; University of Alberta, Canada

## Abstract

**Objective:**

To assess the reliability of contractile properties of the knee extensor muscles in 23 individuals with post-polio syndrome (PPS) and 18 age-matched healthy individuals.

**Methods:**

Contractile properties of the knee extensors were assessed from repeated electrically evoked contractions on 2 separate days, with the use of a fixed dynamometer. Reliability was determined for fatigue resistance, rate of torque development (MRTD), and early and late relaxation time (RT50 and RT25), using the intraclass correlation coefficient (ICC) and standard error of measurement (SEM, expressed as % of the mean).

**Results:**

In both groups, reliability for fatigue resistance was good, with high ICCs (>0.90) and small SEM values (PPS: 7.1%, healthy individuals: 7.0%). Reliability for contractile speed indices varied, with the best values found for RT50 (ICCs>0.82, SEM values <2.8%). We found no systematic differences between test and retest occasions, except for RT50 in healthy subjects (*p* = 0.016).

**Conclusions:**

In PPS and healthy individuals, the reliability of fatigue resistance, as obtained from electrically evoked contractions is high. The reliability of contractile speed is only moderate, except for RT50 in PPS, demonstrating high reliability.

**Significance:**

This was the first study to examine the reliability of electrically evoked contractile properties in individuals with PPS. Our results demonstrate its potential to study mechanisms underlying muscle fatigue in PPS and to evaluate changes in contractile properties over time in response to interventions or from natural course.

## Introduction

Postpoliomyelitis syndrome (PPS) is a complex of late onset neuromuscular symptoms with new or increased muscle weakness and muscle fatigability as key symptoms [1]. PPS often affects the lower limbs, and many individuals experience increased difficulty with walking, standing, climbing stairs and other activities of sustained endurance [2]–[4]. Therefore, interventions aimed at reducing lower limb muscle fatigue are important components in the management of PPS [5]–[7].

Muscle fatigue depends on several factors that may reside in the brain or spinal cord (central fatigue), and/or in the muscles themselves (peripheral fatigue) [8]. Since previous studies suggest that the contribution of central fatigue in PPS is only limited, the focus is on peripheral fatigue [6], [9]. Peripheral fatigue is largely determined by the muscle's aerobic muscle capacity and fiber type composition, and investigation of contractile properties can help to understand the origin of fatigue [10], [11]. Two fundamental contractile properties are the resistance to fatigue and contractile speed. Both properties can be investigated by electrically evoked muscle contractions.

Despite recent findings indicating that resistance to fatigue and contractile speed in individuals with PPS do not seem to differ from age-matched healthy individuals, a marked variability was observed, expressed by the large SD, underscoring the heterogeneity in contractile properties between individuals [12]. This implies that contractile functioning is impaired in part of the individuals with PPS, and intervention programs aimed at reducing muscle fatigue in this subgroup might therefore be useful. Obviously, the application of measurements of contractile properties, for example to evaluate changes following an intervention, requires information about the reliability.

Surprisingly, despite the frequent use of electrically evoked muscle contractions to assess fatigue resistance and contractile speed in healthy subjects and individuals with impaired neuromuscular function [13] only limited information is available about the reliability of these measures. Based on studies containing small numbers of healthy subjects and individuals with spinal cord injury (SCI), reliability seems acceptable [14]–[18]. However, variability may differ between study populations [19], requiring the reliability to be specifically established in individuals with PPS. Furthermore, most studies either addressed reliability with the intraclass correlation coefficient [16], [18], or with parameters of measurement error [14]. Yet, to cover reliability in full, information about both these aspects is required [20], [21].

The purpose of our study was to establish the reliability of fatigue resistance and contractile speed indices of the knee extensor muscles obtained from electrically evoked contractions in individuals with PPS and in healthy individuals. We hypothesized that the reliability of fatigue resistance and contractile speed indices would be high in both groups.

## Methods

### Subjects

Subjects were recruited from the Dutch expert center for polio survivors of the Academic Medical Center in Amsterdam and were all diagnosed with PPS according to the criteria as published by the March of Dimes [1]. They all had a confirmed history of acute poliomyelitis affecting the lower limbs, new symptoms after a period of functional stability, and no other diseases that could explain their reduced muscle strength. Furthermore, subjects were capable of walking with or without walking aids, and had minimum knee extensor strength of at least 30 Newton meter (Nm) in one leg, which was assumed to represent minimal muscle strength for functional use. Thirteen subjects performed the measurements as part of an ongoing clinical trial [22], and the remaining subjects responded to an invitation after chart review for eligibility. In addition, healthy individuals (matched for age and gender) who never had polio or any other neurological disease served as controls. Control subjects were employees of the university or volunteers who had responded to a recruitment advertisement. The study was approved by the medical ethics committee of the Academic Medical Center (University of Amsterdam, The Netherlands) and written informed consent was obtained from all subjects before inclusion.

### Instrumentation

Contractile properties of the knee extensor muscles were assessed from isometric contractions with the knee and hip angle set at 60° and 80° (0° = full extension), respectively, with the use of a specially designed fixed dynamometer. The subjects' upper body and pelvis were restrained to the dynamometer chair with adjustable belts, to prevent the hip from extending during testing. The lower leg was tightly strapped to a lever arm, immediately proximal to the malleoli. The torque applied by the knee extensor muscles was displayed on a screen, digitized (1000 HZ), and stored on disk for off-line analysis. In individuals with PPS, measurements were performed on the leg subjects felt was limiting performance during daily life activities the most. However, if knee extensor strength in this leg was less than 30 Nm, measurements were performed on the other leg. In healthy subjects, the leg that was measured was selected randomly.

Electrical stimulation of the knee extensor muscles was delivered through two self-adhesive surface electrodes (8×13 cm, Schwa-medico, Leusden, The Netherlands). The cathode was placed on the midline of the thigh, 15 cm distal to the anterior superior iliac spine, and the anode was positioned 8 cm proximal to the superior border of the patella. A custom-made software program controlled the frequency and number of square-wave pulses (200 µs), which were delivered by a constant-current high voltage stimulator (model DS7H, Digitimer Ltd., Welwyn Garden City, UK).

### Procedure

Subjects were tested on two different occasions, using exactly the same protocol, with a maximum of three weeks between the two occasions (median interval: 7 days). Test (T1) and retest (T2) measures were performed at the same time of the day, and the same trained researcher performed all measurements and analyses. After anthropometrics were taken, subjects were asked to perform three maximal voluntary isometric knee extensions. Subjects received visual feedback regarding the torque they produced, and were verbally encouraged to exert maximal isometric torque for approximately 3 s, with 1 min of rest between contractions. The highest torque was taken as the maximal voluntary torque (MVT). Subsequently, electrical bursts (150 Hz, to ensure maximal activation) of 1 s duration were delivered to the muscle with increasing current, until ∼30% of MVT was reached, sufficient to activate a representative part of the muscle mass [19]. After a five-minute resting period, fatigue resistance was determined by a series of electrically evoked isometric tetanic contractions (50 Hz) of 1 s duration and 1 s of rest in between, for a period of five minutes (150 contractions). At this 50 Hz frequency, considerable torque is generated [12], and high frequency fatigue is prevented [8]. The latter was confirmed by visual inspection of the raw data.

### Data analysis

Off-line analysis of torque records was performed using a custom-written Matlab script (version R2007a, The Mathworks Inc., S. Natick, MA, USA). Each torque signal was filtered with a low-pass fourth order Butterworth filter with a 50 Hz cut-off frequency. Fatigue resistance was expressed as the torque at the end of the fatigue protocol divided by that at the start of the protocol: fatigue resistance [% torque remaining]  =  (average last 30 contractions/first contraction) x 100%) [16], [23].

In addition to fatigue resistance and the maximal voluntary torque (MVT), we established three indices of contractile speed: i) the maximal rate of torque development, calculated as the highest value of the differentiated torque signal, expressed relative to the highest torque during that contraction (MRTD [s^−1^]) [24]; ii) early half-relaxation time, defined as the time taken for torque to decline from the value at the end of stimulation to 50% of that value (RT50 [ms]) [8]; and iii) late half-relaxation time, i.e. the time needed for torque to fall from 50% to 25% (RT25 [ms]) [8]. Contractile speed indices were determined from the first contraction of the fatigue protocol.

### Statistical analysis

Descriptive data were expressed as mean and standard deviation (demographic data) or as median and range (polio characteristics). Differences between individuals with PPS and healthy subjects regarding demographic data were analyzed with the Student's *t* test for normally distributed data; in case of non-normally distributed data, the Mann-Whitney *U* test was used. Dichotomized variables were analyzed with Fisher's exact test.

Test-retest reliability [25] was assessed with the intraclass correlation coefficient (ICC_2,1_) and the 95% confidence intervals (CI), by use of a random effects two-way analysis of variance (ANOVA). A lower CI limit of the ICC≥.75 was considered to indicate excellent reliability [26]. Systematic changes between test occasions together with the 95% CI were analyzed with paired *t* tests. A systematic change is a nonrandom change that occurs if the subject systematically performs better (or worse) on the second test occasion as a result of, for example, a change in behavior or a learning effect.

Measurement error [25] was calculated according to the equations proposed by Euser et al. [27] (see Appendix S1), which is based on ANOVA. Measurement error was expressed either in the actual units of the measurements (standard error of measurement (SEM) and absolute limits of agreement (ALoA)) or as a proportion of the measured values (coefficient of variation based on log transformed variables (CV) and ratio limits of agreement (RLoA)). The CV and RLoA were used in case of heteroscedasticity, implying that measurement error was dependent of the parameter mean. Data was denoted heteroscedastic if the correlation coefficient (Kendall's τ) between the absolute differences and the corresponding means was >0.1 [28]. Bland-Altman graphs were plotted for visual interpretation of the data.

To determine the influence of measurement error on sample size estimation for an effect study comparing 2 independent groups, we calculated the minimal number of subjects per group needed (n) to find a significant difference in change of fatigue resistance from n>2(Z_α_+Z_β_)^2^ σ^2^/δ^2^, with Z values based on tables of standard normal curves (Z_α_+Z_β_ = 3.242 for a = .05 and b = .10), σ as the standard deviation of the difference, and δ as the minimal difference in effect that is considered of clinical interest [29]. Because it is unknown what changes are considered clinically relevant, we included three sample size estimations based on different change scores (i.e. 10%, 15% and 20%).

For all tests, the significance level was set at *p*<.05. Statistical analyses were performed with the SPSS statistical software package (version 20.0.0.1, IBM Company, Armonk, NY, USA).

## Results

Twenty-nine individuals with PPS and 20 healthy subjects participated. Two healthy subjects and 4 PPS subjects prematurely aborted the measurements due to discomfort of the electrical stimulation. Furthermore, in 2 individuals with PPS targeted stimulated torque levels were not achieved despite high current levels. Therefore, analyses were performed on 23 individuals with PPS (9 men) and 18 healthy individuals (6 men). Characteristics of both groups are presented in Table 1. None of the demographic variables presented in Table 1 differed significantly between both groups (*p*>0.072).

**Table 1:** Subject characteristics.

	PPS (*n* = 23)	Control (*n* = 18)
*Demographic data*		
Gender (male/female)	9/14	6/12
Age (yrs)	59.9±6.3	58.5±6.8
Weight (kg)	77.3±12.7	74.7±8.5
BMI (kg/m^2^)	26.4±2.8	24.7±2.9
*Polio characteristics*		
Age at acute polio (yrs)	1.0 (0–9)	-
Time since new symptoms (yrs)	15 (3–33)	-
Present walking distance*	3 (2–4)	-
Measured leg (most/less affected)	13/10	-

Values for demographic data are mean ± SD; values for polio characteristics are median (range).

*Abbreviations*: PPS, post-polio syndrome; BMI, body mass index.

*Walking distance was defined as the daily distance walked and was classified in 4 categories: 1 (indoors only), 2 (around the house), 3 (seldom >1 km), and 4 (regularly >1 km).

The mean difference in fatigue resistance between both tests was −0.28% and 2.06% for individuals with PPS and healthy subjects, respectively, with no systematic differences observed (Table 2). The ICC values and corresponding 95% CIs were high and comparable in PPS (ICC = 0.90 [95% CI: 0.77–0.96]) and healthy subjects (0.91 [0.77–0.97]).

**Table 2:** Results on test and retest for individuals with PPS and healthy subjects.

	n	Test (T1)	Retest (T2)	Δ T2–T1	95% CI_Δ_	ICC_2,1_ (95% CI_ICC_)
**Fatigue resistance**						
*Fatigue resistance (% torque remaining)*						
PPS	19	48.0±10.3	47.7±11.6	−0.28±4.90	−2.64–2.08	0.90 (0.77–0.96)
Control	16	51.6±12.9	53.7±12.2	2.06±4.98	−0.60–4.71	0.91 (0.77–0.97)
**MVT and contractile speed indices**						
*MVT (Nm)*						
PPS	23	116.8±47.3	114.2±48.9	−2.54±12.51	−7.95–2.86	0.97 (0.92–0.99)
Control	18	177.7±33.8	176.1±32.7	−1.67±9.10	−6.20–2.85	0.96 (0.91–0.99)
*MRTD (s^−1^)*						
PPS	23	15.4±7.9	15.0±6.4	−0.35±6.55	−3.18–2.48	0.59 (0.24–0.81)
Control	18	16.6±9.4	15.2±8.3	−1.39±6.11	−4.43–1.65	0.76 (0.48–0.90)
*RT50 (ms)*						
PPS	23	125.0±8.3	125.2±8.4	0.22±3.92	−1.48–1.91	0.89 (0.77–0.95)
Control	18	122.6±8.2	125.2±7.6	2.67±4.23	0.56–4.77	0.82 (0.49–0.93)
*RT25 (ms)*						
PPS	23	28.0±3.5	28.9±3.5	0.87±4.69	−1.16–2.90	0.10 (−0.33–0.48)
Control	18	31.9±6.1	30.6±5.1	−1.33±4.80	−3.72–1.06	0.63 (0.25–0.84)

The score on the two visits and the difference (Δ) are presented as mean ± SD.

Missing data are due to the difficulty of some subjects to relax the leg during the stimulated protocol, resulting in unworkable signals, and leading to different numbers of observations for different parameters.

*Abbreviations*: ICC, intraclass correlation coefficient; CI, confidence interval; PPS, post-polio syndrome; RT50, relaxation time 50%; MVT, maximal voluntary torque; Nm, Newton meter; MRTD, maximal rate of torque development; RT25, relaxation time 25%; ms, milliseconds.

For contractile speed indices and MVT, differences between the means of outcomes on the two occasions can be found in Table 2. Only for RT50 in healthy subjects, a systematic difference between test scores was observed (2.67 ms, *p* = 0.016).

Test-retest reliability varied between outcomes, both in PPS and in healthy individuals. For example, in individuals with PPS, ICCs ranged from 0.10 for RT25 [95% CI: −0.33–0.48] to 0.97 for MVT [0.92–0.99].

**Figure 1:**
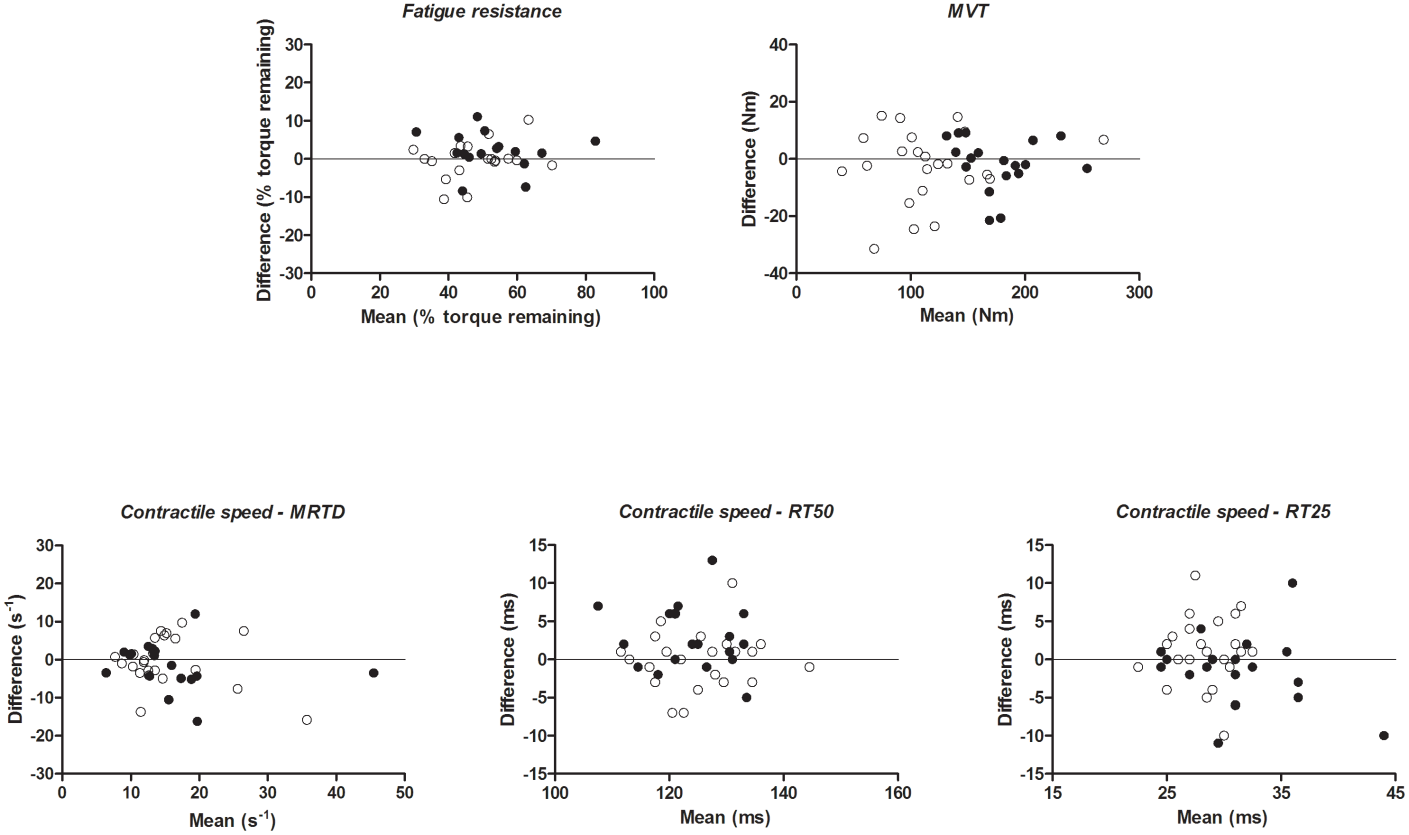
The Bland-Altman graphs with the differences between test sessions 2 and 1 (test 2 minus test 1) plotted against the means of the 2 test sessions for the five studied variables, for patients with PPS (o) and healthy subjects (•).

Table 3 and Figure 1 present the results on measurement error and the Bland-Altman graphs for all variables. In PPS, the SEM for fatigue resistance was 3.38% and the ALoA ranged from −9.9 to 9.3%, indicating a real difference at individual level of 9.6%. This represents 20% of the mean fatigue resistance of T1 and T2 (i.e. 9.6%/47.9%). Similar results were found for healthy subjects. In both groups, measurement error for RT50 was much smaller compared to the values found for the other indices of contractile speed and for MVT (Table 3).

**Table 3:** Results for measurement error for individuals with PPS and healthy subjects.

	*τ*-correlation coefficient	SEM (% of mean)	ALoA	CV	RLoA
**Fatigue resistance**					
*Fatigue resistance (% torque remaining)*					
PPS	−0.065 (*p* = 0.700)	3.38 (7.1%)	−9.9–9.3	-	-
Control	−0.059 (*p* = 0.752)	3.70 (7.0%)	−7.7–11.8	-	-
**MVT and contractile speed indices**					
*MVT (Nm)*					
PPS	−0.138 (*p* = 0.355)	8.83 (7.6%)	−27.1–22.0	-	-
Control	−0.033 (*p* = 0.850)	6.36 (3.6%)	−19.5–16.2	-	-
*MRTD (s^−1^)*					
PPS	0.518* (*p* = 0.001)	-	-	30.0%	0.42–2.28
Control	0.399* (*p* = 0.021)	-	-	26.8%	0.44–1.96
*RT50 (ms)*					
PPS	0.009 (*p* = 0.957)	2.71 (2.2%)	−7.5–7.9	-	-
Control	−0.042 (*p* = 0.816)	3.46 (2.8%)	−5.6–11.0	-	-
*RT25 (ms)*					
PPS	0.123 (*p* = 0.437)	-	-	11.6%	0.75–1.42
Control	0.338 (*p* = 0.063)	-	-	10.2%	0.73–1.27

*Abbreviations*: SEM, standard error of measurement; ALoA, absolute limits of agreement; CV, coefficient of variation; RLoA, ratio limits of agreement; PPS, post-polio syndrome; RT50, relaxation time 50%; MVT, maximal voluntary torque; Nm, Newton meter; MRTD, maximal rate of torque development; RT25, relaxation time 25%; ms, milliseconds.

*Significant correlation (*p*<.05, two tailed) using Kendall's *τ*-correlation coefficient.

Table 4 shows the results of the sample size estimations based on different change scores. For example, assuming that a 10% change in fatigue resistance between two groups of individuals with PPS is clinical relevant, the total number of subjects for an intervention study would be 44 (i.e. 2 groups of 22 subjects).

**Table 4:** Sample size estimation for an effect study to detect improvement in fatigue resistance between 2 independent groups of individuals with PPS.

	Minimal number of subjects per group needed (n)
**Assumed clinically relevant change**	
10%	22
15%	10
20%	6

We calculated the minimal number of subjects per group (n) needed to find a significant change of fatigue resistance from n>2(Zα+Zβ)^2^ σ^2^/δ^2^, with Z values based on tables of standard normal curves (Zα+Zβ = 3.242 for a = .05 and b = .10), σ as the standard deviation of the difference (4.90%), and δ as the minimal difference in effect that is considered of clinical interest. Because it is unknown what change is considered clinically relevant we estimated sample size based on different change scores (i.e. 10%, 15% and 20%).

## Discussion

To our knowledge, this is the first study that assessed both test-retest reliability and measurement error of contractile properties obtained from electrically evoked contractions in a patient population. Our results indicate that both in individuals with PPS and in healthy individuals, the reliability of fatigue resistance is high and sensitive to detect changes at group level. The reliability of contractile speed indices is only moderate, except for RT50 in PPS, demonstrating high reliability. Considering these results, the assessment of fatigue resistance and RT50 seems suitable for clinical use, for example to evaluate changes in contractile properties following interventions aimed at reducing muscle fatigue in PPS.

In the present study we found high ICC values for fatigue resistance and RT50, both in individuals with PPS and healthy persons, indicating adequate test-retest reliability. Our results with respect to fatigue resistance are similar to those reported in previous studies in healthy subjects (with ICCs ranging from 0.78 to 0.92) [15]–[18]. Results regarding contractile speed indices cannot be directly compared, because this was the first study that assessed the test-retest reliability of these measures, based on the ICC. We found that test-retest reliability of RT50 is better than the test-retest reliability of the two other indices of contractile speed, both in individuals with PPS and in healthy persons. Considering these results, it may be hypothesized that late half-relaxation time (RT25) and rate of torque development (MRTD) are influenced more by additional voluntary muscle activity that possibly occurred during the stimulated contractions than early half-relaxation time (RT50) [30]. However, there is no evidence to support this hypothesis. Taken together, the high ICCs as found in the present and in previous studies indicate that the test-retest reliability of fatigue resistance and RT50 to discriminate between persons with different levels of muscle fatigue is adequate.

Adequate reliability in terms of a high ICC does not directly imply that the outcome measure is suitable for clinical use. In addition, measurement error should be small enough to detect clinically relevant changes. In the present study, measurement error for fatigue resistance (SEM<3.70%, representing 7.0% of the mean) and RT50 (SEM<3.46 ms, 2.8% of the mean) were low. These values are in agreement with those established in other patient groups (e.g. spinal cord injury) [14], and, moreover, they indicate that measurements can be made reliably for a group of individuals with PPS. Unfortunately, it is not known what changes in contractile properties of the knee extensor muscles in individuals with PPS are considered clinically relevant, and future research is therefore required. Nevertheless to interpret the measurement error values found in our study, we related our data to previously established training effects for contractile properties. We found two studies reporting on changes in volitional fatigue resistance of the knee extensors following exercise training in PPS, ranging from 10 to 21% [5], [31]. Combined with the sample size estimations, based on the current reliability data, this indicates that feasible sample sizes are required to detect improvement in fatigue resistance between two independent groups of individuals with PPS.

While our results suggest that electrically evoked contractions can be used to detect changes in fatigue resistance and RT50 at group level, we consider this method insufficiently sensitive to detect changes in single individuals. For example, based on the limits of agreement, it appeared that the relative change in fatigue resistance should exceed 20% to indicate a real change at individual level. Only when larger therapeutic benefits are expected, this outcome measure is useful for this purpose.

## Limitations

Our study is limited by the relatively small sample size, restricting generalizability of our results to the population of individuals with PPS and healthy individuals in general. Another limitation is that we performed measurements solely on the knee extensor muscles and not on other muscle groups. Although from the literature it is known that the effects of polio are widespread and not necessarily restricted to one muscle group [32], we have chosen to investigate this muscle group. This because muscle weakness in PPS often affects the lower limbs, and also measurements can be easily performed on this muscle group that is of major importance during locomotion-related activities [33]. Nonetheless, even though we consider it legitimate that we performed measurements only on the knee extensor muscles, it must be realized that results cannot simply be generalized to other muscle groups.

## Conclusions

Both in individuals with PPS and in healthy individuals, the reliability of fatigue resistance, as obtained from electrically evoked contractions of the knee extensor muscles is high. The reliability of contractile speed indices is only moderate, except for RT50 in PPS, demonstrating high reliability. Considering these results, the assessment of contractile properties in PPS is sufficiently reliable to identify those patients with impaired contractile functioning of their knee extensor muscles, and, accordingly, to evaluate changes over time or following interventions in this patient group. Based on its potential in PPS, future research may also focus on the feasibility of this method in other slowly progressive neuromuscular diseases where muscle fatigue is a major problem.

## Supporting Information

Appendix S1 **Reliability measures regarding measurement error.** Measurement error was assessed based on a two-way analysis of variance (ANOVA). The three components of variance that were estimated with this analysis included the inter-subject variance (var_s_), the variance related the repeated sessions (occasion variance, var_o_), and the error variance (var_e_). These latter two were used to calculate the standard error of measurement (SEM) and coefficient of variation (CV). Abbreviations: SEM, standard error of measurement; var_o_: occasion variance; var_e_: error variance; CV, coefficient of variation; ALoA, absolute limits of agreement; SD: standard deviation; RLoA, ratio limits of agreement.
(DOC)

Dataset S1 **Dataset used for statistical analysis.**
(SAV)
