# Supplementary material for: Reliability of Contractile Properties of the Knee Extensor Muscles in Individuals with Post-Polio Syndrome
Source: PLoS One. 2014 Jul 14;9(7):e101660. doi: 10.1371/journal.pone.0101660 (PMC4096590; doi:10.1371/journal.pone.0101660)
Supplement: Appendix S1 — Reliability measures regarding measurement error. Measurement error was assessed based on a two-way analysis of variance (ANOVA). The three components of variance that were estimated with this analysis included the inter-subject variance (vars), the variance related the repeated sessions (occasion variance, varo), and the error variance (vare). These latter two were used to calculate the standard error of measurement (SEM) and coefficient of variation (CV). Abbreviations: SEM, standard error of measurement; varo: occasion variance; vare: error variance; CV, coefficient of variation; ALoA, absolute limits of agreement; SD: standard deviation; RLoA, ratio limits of agreement. (DOC) [file pone.0101660.s001.doc]

**Appendix S1**

Reliability measures regarding measurement error.

Measurement error was assessed based on a two-way analysis of variance (ANOVA). The three components of variance that were estimated with this analysis included the inter-subject variance (var_s_),_the variance related the repeated sessions (occasion variance, var_o_), and the error variance (var_e_). These latter two were used to calculate the standard error of measurement (SEM) and coefficient of variation (CV).

SEM $\sqrt{\left(\text{var}_{\text{o}}+\text{var}_{\text{e}}\right)}$

Where $\sqrt{\left(\text{var}_{\text{o}}+\text{var}_{\text{e}}\right)}$ is the spread of original measurements from different occasions.

CV $100\%\times\ln\left(10\right)\sqrt{\left(\text{var}_{\text{o}}+\text{var}_{\text{e}}\right)}$

Where $\sqrt{\left(\text{var}_{\text{o}}+\text{var}_{\text{e}}\right)}$ is the spread of the log-transformed measurements from different occasions. The ln(10) is used, because we considered the 10-log transformation.

ALoA Mean difference ± $1.96\times$SD difference

Where SD difference is the standard deviation of the differences between the original measurements.

RLoA Antilog (Mean difference log ± $1.96\times$SD difference log)

Where mean difference log and SD difference log are the mean and standard deviation of the differences between the log-transformed measurements.

*Abbreviations*: SEM, standard error of measurement; var_o_: occasion variance; var_e_: error variance; CV, coefficient of variation; ALoA, absolute limits of agreement; SD: standard deviation; RLoA, ratio limits of agreement.
